# Supplementary material for: The Bacterial Genomic Context of Highly Trimethoprim-Resistant DfrB Dihydrofolate Reductases Highlights an Emerging Threat to Public Health
Source: Antibiotics (Basel). 2021 Apr 13;10(4):433. doi: 10.3390/antibiotics10040433 (PMC8103504; doi:10.3390/antibiotics10040433)
Supplement: Supplementary file 1 [file antibiotics-10-00433-s001.pdf]

Supporting Information

The genomic context of highly trimethoprim-resistant DfrB dihydrofolate reductases highlights an emerging threat to public health

Claudèle Lemay-St-Denis[1,2,3], Sarah-Slim Diwan[1,2,3] and Joelle N. Pelletier[1,2,3,4]

[1] Department of Biochemistry and Molecular Medecine, Université de Montréal, Montréal, Québec, Canada

[2] PROTEO, the Québec Network for Research on Protein, Function, Engineering and Applications, Qc, Canada

[3] CGCC, Center in Green Chemistry and Catalysis, Montréal H3A 0B8, Qc, Canada

[4] Chemistry Department, Université de Montréal, Montréal, Québec, Canada

Table S1. Protein sequence identity of the ten members of the DfrB family [a]

	DfrB1	DfrB2	DfrB3	DfrB4	DfrB5	DfrB6	DfrB7	DfrB9	DfrB10	DfrB11
DfrB1	100%	78%	78%	77%	88%	87%	88%	77%	82%	78%
DfrB2	17	100%	86%	74%	79%	82%	78%	84%	92%	88%
DfrB3	17	11	100%	79%	82%	83%	79%	85%	92%	90%
DfrB4	18	20	16	100%	77%	77%	76%	74%	77%	79%
DfrB5	9	16	14	18	100%	91%	94%	77%	83%	81%
DfrB6	10	14	13	18	7	100%	91%	79%	86%	83%
DfrB7	9	17	16	19	5	7	100%	76%	82%	81%
DfrB9	18	12	12	20	18	16	19	100%	88%	86%
DfrB10	14	6	6	16	13	11	14	9	100%	92%
DfrB11	17	9	8	18	15	13	15	11	6	100%

[a] Values in percentage correspond to protein sequence identity values; values in grey represent the number of mutations between sequences.

Table S2. DfrB sequences

Gene	Genbank ID	Gene sequence	Protein sequence
dfrb1	KAB1871659.1	ATGGAACGAAGTAGCAATGAAGTCAGTAATC<br>CAGTTGCTGGCAATTTTGTATTCCCATCGAACG<br>CCACGTTTGGTATGGGAGATCGCGTGCGCAAG<br>AAATCCGGCGCCGCCTGGCAAGGTCAGATTGT<br>CGGGTGGTACTGCACAAATTTGACCCCCGAAG<br>GCTACGCCGTCGAGTCTGAGGCTCACCCAGGC<br>TCAGTACAGATTTATCCTGTTGCGGCGCTTGA<br>ACGCATCAACTGA	MERSSNEVSNPV<br>AGNFVFPSNATF<br>GMGDRVRKKSG<br>AAWQGQIVGW<br>YCTNLTPEGYAV<br>ESEAHPGSVQIY<br>PVAALERIN
dfrb2	FAA00064.1	ATGGGTCAAAGTAGCGATGAAGCCAACGCTC<br>CCGTTGCAGGGCAGTTTGCGCTTCCCCTGAGT<br>GCCACCTTTGGCTTAGGGGATCGCGTACGCAA<br>GAAATCTGGTGCCGCTTGGCAGGGTCAAGTCG<br>TCGGTTGGTATTGCACAAAACTCACTCCTGAA<br>GGCTATGCGGTCGAGTCCGAATCCCACCCAGG	MGQSSDEANAP<br>VAGQFALPLSAT<br>FGLGDRVRKKS<br>GAAWQGQVVG<br>WYCTKLTPEGY<br>AVESESHPGSVQ<br>IYPVAALERVA

		CTCAGTGCAAATTTATCCTGTGGCTGCACTTGA<br>ACGTGTGGCCTAA	
dfrb3	ACR57831.1	ATGGACCAACACAACAATGGAGTCAGTACTC<br>TAGTTGCTGGCCAGTTTGCGCTCCCATCGCAC<br>GCCACGTTTGGCCTGGGAGATCGCGTGCGCAA<br>GAAATCTGGCGCCGCTTGGCAGGGTCAAGTTG<br>TCGGGTGGTACTGCACAAAACTGACCCCTGAA<br>GGCTATGCCGTCGAGTCCGAGTCTCACCCCGG<br>TTCAGTACAGATTTATCCTGTGGCTGCGCTTGA<br>ACGCGTGGCCTGA	MDQHNNGVSTL<br>VAGQFALPSHA<br>TFGLGDRVRKKS<br>GAAWQGQVVG<br>WYCTKLTPEGY<br>AVESESHPGSVQ<br>IYPVAALERVA
dfrb4	ALF62656.1[a]	ATGAATGAAGGAAAAAATGAGGTCAGTACTT<br>CAGCTGCTGGCCGGTTCGCATTCCCATCAAAC<br>GCCACGTTTGCCTTGGGGGATCGCGTACGCAA<br>GAAGTCTGGCGCTGCTTGGCAGGGGCGCATTG<br>TCGGGTGGTACTGCACAACACTTACCCCTGAA<br>GGCTACGCCGTCGAGTCCGAATCTCACCCAGG<br>CTCAGTCCAGATTTATCCCATGACTGCGCTTG<br>AACGGGTGGCCTGA	MNEGKNEVSTS<br>AAGRFAFPSNAT<br>FALGDRVRKKS<br>GAAWQGRIVG<br>WYCTTLTPEGYA<br>VESESHPGSVQIY<br>PMTALERVA
dfrb5	AAX46054.1	ATGGACCAAGGCAGAAGTGAAGTCAGTAATC<br>CAGTTGCTGGCCAGTTTGCGTTCCCTTCAAAC<br>GCCGCGTTCGGAATGGGAGATCGCGTGCGCA<br>AGAAATCTGGCGCCGCTTGGCAAGGCCAGATT<br>GTCGGGTGGTACTGCACAAAATTGACCCCTGA<br>AGGGTACGCTGTCGAGTCTGAGGCTCACCCTG<br>GCTCGGTACAGATTTATCCTGTTGCGGCACTG<br>GAACGCATCAACTGA	MDQGRSEVSNP<br>VAGQFAFPSNA<br>AFGMGDRVRKK<br>SGAAWQGQIVG<br>WYCTKLTPEGY<br>AVESEAHPGSV<br>QIYPVAALERIN
dfrb6	ADO00942.1	ATGGACCAAGGTAGCAATGAAGTCATTAATC<br>CAGTCGCTGGCCAGTTTGCGTCCCCATCGAAC<br>GCCACGTTTGGTATGGGAGATCGCGTGCGCAA<br>GAAATCTGGCGCCGCCTGGCAAGGTCAGATTG<br>TCGGGTGGTACAGCACAAAGTTGACCCCTGA<br>AGGCTACGCTGTCGAGTCTGAGGCTCACCCTG<br>GCTCGGTGCAGATTTATCCTGTTGCCGCGCTTG<br>AACGCGTCAACTGA	MDQGSNEVINP<br>VAGQFASPSNA<br>TFGMGDRVRKK<br>SGAAWQGQIVG<br>WYSTKLTPEGYA<br>VESEAHPGSVQI<br>YPVAALERVN
dfrb7	ADB54781.1	ATGGACCAAGGTAGCAATGAAGTCGGTAATC<br>CAGTTGCGGGCCAGTTTTCGTTCCCATCGAAC<br>GCCGCGTTTAGTATGGGAGATCGCGTGCGCAA<br>GAAATCGGGCGCCGCTTGGCAAGGTCAGATT<br>GTCGGGTGGTACTGCACAAAGTTGACCCCTGA<br>AGGCTACGCTGTCGAGTCTGAGGCTCACCCTG<br>GCTCGGTACAGATTTATCCTGTTGCGGCGCTTG<br>AACGCATCAACGGAGTTCAAGGTTGA	MDQGSNEVGNP<br>VAGQFSFPSNAA<br>FSMGDRVRKKS<br>GAAWQGQIVG<br>WYCTKLTPEGY<br>AVESEAHPGSV<br>QIYPVAALERIN<br>GVQG
dfrb9	AGM20434.1	ATGAATCAAAGTAGCAATTGCATCAGCACTCC<br>AGTTGTTGGACAGTTTGCGCTGCCATTTCAACC<br>CACGTTCGGCCTGGGAGATCGCGTACGCAAG<br>AAGTCTGGCGCCGCTTGGCAAGGTAAAGTTGT<br>CGGCTGGTACTGCACAAAATTAACCCCTGAAG<br>GCTACGCGGTCGAGTCCGAAGCTCATCCAGGC<br>TCAGTGCAGATTTATCCTGTGGCTGCGCTTGA<br>ACGCGTGGCCTAA	MNQSSNCISTPV<br>VGQFALPFQPTF<br>GLGDRVRKKSG<br>AAWQGKVVGW<br>YCTKLTPEGYAV<br>ESEAHPGSVQIY<br>PVAALERVA
dfrb10	ALZ46148.1	ATGGATCAAAGTAGCAATGAAGTCAGCACTC<br>CAGTTGCTGGCCAGTTTGCGCTCCCATTGCGC	MDQSSNEVSTPV<br>AGQFALPLRATF

		GCCACGTTTGGCCTGGGAGATCGCGTACGCAA<br>GAAATCTGGCGCCGCTTGGCAAGGTCAAGTTG<br>TCGGCTGGTACTGCACAAAACTGACCCCTGAA<br>GGCTATGCAGTCGAGTCCGAGTCTCACCCAGG<br>CTCAGTACAGATTTATCCTGTGGCTGCGCTTGA<br>ACGCGTGGCCTAA	GLGDRVRKKSG<br>AAWQGQVVGW<br>YCTKLTPEGYAV<br>ESESHPGSVQIYP<br>VAALERVA
dfrb11	PKO69073.1	ATGGATCAAAGTAGTAAAGAGGTTGGCACTC<br>CCGTTGTTGGCCAGTTTGCACTCCCGTCGCAC<br>GCCACGTTTGGCCTTGGAGACCGCGTTCGCAA<br>GAAATCGGGCGCCGCTTGGCAGGGTCAAGTT<br>GTGGGCTGGTATTGCACAAAGCTGACCCCTGA<br>AGGCTATGCCGTCGAGTCCGAGTCTCACCCAG<br>GCTCGGTACAAATTTATCCAGTGAATGCGCTT<br>GAACGCGTGGCCTGA	MDQSSKEVGTP<br>VVGQFALPSHA<br>TFGLGDRVRKKS<br>GAAWQGQVVG<br>WYCTKLTPEGY<br>AVESESHPGSVQ<br>IYPVNALERVA

[a] This sequence differs from CARD's reference sequence ABY55281.1 at the highlighted region (here Gly instead of Asp). Since we previously characterized DfrB4 using ALF62656.1 sequence [1], we used it in this study.

1. Toulouse, J.L.; Shi, G.; Lemay-St-Denis, C.; Ebert, M.C.C.J.C.; Deon, D.; Gagnon, M.; Ruediger, E.; Saint-Jacques, K.; Forge, D.; Vanden Eynde, J.J.; et al. Dual-Target Inhibitors of the Folate Pathway Inhibit Intrinsically Trimethoprim-Resistant DfrB Dihydrofolate Reductases. *ACS Med. Chem. Lett.* **2020**, acsmedchemlett.0c00393, doi:10.1021/acsmedchemlett.0c00393.
